# Oral and systemic health: is there a “new” link with COVID-19?

**DOI:** 10.1007/s00784-023-04950-2

**Published:** 2023-04-20

**Authors:** David Herrera, Jorge Serrano, Silvia Roldán, Bettina Alonso, Mariano Sanz

**Affiliations:** ETEP (Etiology and Therapy of Periodontal and Peri-implant Diseases) Research Group, University Complutense of Madrid, Madrid, Spain

**Keywords:** SARS-CoV-2, COVID-19, Periodontitis, Comorbidities, Oral health

## Abstract

**Objectives:**

The objective of the present narrative review was to evaluate the evidence of a possible association between periodontitis and COVID-19, and its biological plausibility, using as models the potential associations with cardiovascular diseases, diabetes, and some respiratory diseases.

**Methods:**

A recent systematic review was used as main reference to explore the associations of periodontitis with different respiratory diseases, including COVID-19, following two focussed questions: a PECOS question, aimed to explore epidemiological evidence, and a PICOS question, designed to explore the evidence derived from intervention studies. In addition to that evidence, other relevant scientific documents, including consensus papers, were carefully selected and appraised.

**Findings:**

Convincing evidence was found to support the association of periodontitis and cardiovascular diseases, diabetes, and some respiratory diseases. The biological plausibility behind those associations is based on four factors: (1) bacteraemia of oral bacteria and periodontal pathogens, (2) increased systemic inflammation, (3) common genetic factors, and (4) common environmental risk factors. Limited initial evidence is available to support an association between periodontitis and COVID-19 complications. Among the proposed factors to explain the suggested association, a combination of the previously mentioned factors, plus additional factors related with SARS-CoV-2 characteristics and pathogenicity, has been suggested.

**Conclusions:**

Initial evidence suggests that periodontitis may be associated with a more severe COVID-19 and with a higher risk of death due to COVID-19.

**Clinical relevance:**

Due to the possible association between periodontitis and an increased severity for COVID-19, additional efforts should be made to improve oral and periodontal health, including the promotion of oral healthy habits, such as oral hygiene.

## Introduction

Coronavirus 2 of severe acute respiratory syndrome (SARS-CoV-2), a member of the Coronaviridae family, is the responsible agent of the disease referred as 2019 coronavirus disease (COVID-2019). This disease was first identified in Wuhan (China) in December 2019. The World Health Organization (WHO) declared in March 2020 that SARS-CoV-2 was a global pandemic [1], and subsequently, it spread globally, with 643,875,406 confirmed cases of COVID-19 reported to WHO until December 14, 2022, including 6,630,082 deaths [2].

This highly infectious, spreadable, and dangerous pathogen has caused a huge health, social, and economic impact [3], leading to a worldwide collaborative effort to find an efficient strategy to develop vaccines [4], which have provided an effective immune response, thus safeguarding the community from the virus’ severity [5]. Nevertheless, this viral infection can still present with mild to severe symptomatology, leading in the severe cases to hospitalization due to respiratory distress, including chest pain, shortness of breath accompanied by low blood oxygen, and loss of motor functions. The risk factors associated with this severe systemic presentation in a small proportion of patients infected with SARS-CoV-2 have not been properly identified, although it has been suggested that the presence of other comorbidities, such as hypertension, diabetes, coronary disease, ageing, and obesity, may play a significant role [6].

The role of the oral cavity, as one of the portals of entry of the SARS-CoV-2 virus into the body, and its possible role as a protective/aggravating factor in the infectivity and in the progression of this viral infection were immediately suggested [7], and they have been extensively evaluated. At least, three relevant areas of interest, linking the mouth and COVID-19, have been explored:

- The relevant role of the oral cavity mucosal lining in the transmission and pathogenicity of SARS-CoV-2 [8].
- The possible impact of oral interventions in the transmission of SARS-CoV-2, including the production of aerosols in the dental settings, as well as the possible preventive effect of using of mouth rinses with virucidal action [7].
- The proposed higher risk of periodontitis patients with the onset and severity of COVID-19 [9].

This narrative review has evaluated this third hypothesis, within the context of the well-established associations between periodontitis and different systemic diseases, hypothesizing that the increased chronic systemic inflammation associated with periodontitis is associated to a higher risk of increased severity of COVID-19 in these periodontitis patients. This predicate is supported by the available scientific evidence supporting the relevance of oral health, and specifically of periodontal health, on systemic health [10, 11] and, once again, emphasizes the importance of oral health in the maintenance of an overall systemic health [12, 13].

It was therefore the objective of the present review to evaluate a possible association between periodontitis and COVID-19 severity, and to assess its biological plausibility, using as models the well-established associations with cardiovascular diseases and diabetes, and the potential associations with other respiratory diseases.

## Methods

As main reference for a systematic assessment of the literature, a recently published systematic review from our research group was selected [9]. In this systematic review, the evidence of an association between periodontitis with different respiratory diseases, including COVID-19, was analysed through two focussed questions. The first one, a PECOS question: “in subjects ≥18 years old [population] with periodontitis [exposure] compared with healthy or gingivitis subjects [comparison], what is the prevalence/incidence of COVID-19 [outcome] in cross-sectional, cohort or case-control studies, with a minimum follow-up of 12 months, and a minimum sample of 10 subjects per group [study design]?” The second one, a PICOS question: “in subjects ≥18 years old with periodontitis and COVID-19 [population], which is the effect of periodontal treatment [intervention] when compared with absence of therapy or minimum periodontal treatment [comparison], in terms of changes in the respiratory disease status or surrogate measures of the respiratory disease of interest [outcomes] in randomized clinical trials (RCTs) or controlled clinical trials (CCTs), with a minimum follow-up of 12 months, and a minimum sample of 10 subjects (5 subjects per group) [study design]?” Additional details on the methodology, including the search, can be found in the original publication [9].

In addition to this evidence, systematically retrieved, other relevant scientific documents, including consensus papers, were carefully selected and appraised.

## Findings

### Association of periodontitis and systemic diseases

In a systematic review published in 2016, it was reported that up to 57 systemic conditions have been evaluated (up to May 2015) for their possible association with periodontitis [14]. The most relevant in terms of number of scientific publications were cardiovascular diseases, diabetes, and respiratory diseases, and these were the focus of a recently published consensus [15] of a joint Focused Workshop of the European Federation of Periodontology (EFP) with WONCA Europe, the most important association of family doctors.

Cardiovascular diseases (CVDs) are a group of conditions representing the leading cause of mortality in the world, accounting for 32% of all deaths [16] and 45% of those related with non-communicable diseases (NCDs) [17]. The association between periodontitis and CVDs was first scientifically evaluated in 1989, when a pioneer research from Mattila and co-workers was published [18]. Since then, the available evidence [19] indicates that periodontitis is an independent risk factor for cardiovascular diseases, as concluded in a joint Focused Workshop of the EFP and the World Heart Federation (WHF) in 2019 [11, 20], evaluating these associations and their health-related implications.

Diabetes presents a prevalence, at a worldwide level, of 9.3% (approximately 463 million cases), but an increase is foreseeable, with approximately 578 million cases (10.2% of the population) in 2030 and 10.9% (approximately 700 million) in 2045 [21]. Diabetes, particularly type 2, is one of the main causes of disability and premature mortality (due to the associated complications) [22]. As concluded in the joint Focused Workshop of the EFP and the International Diabetes Federation (IDF) in 2017 [10, 23], a clear bidirectional association of diabetes with periodontitis has been clearly established: periodontitis increases the risk of diabetes onset, poorest glycaemic control, and development of diabetes complications in people with diabetes; and conversely, diabetes increases the risk of periodontitis onset and progression, especially in poorly controlled diabetes patients.

Respiratory diseases include a large variety of very prevalent conditions, some being chronic, as chronic obstructive pulmonary disease (COPD), while others acute, such as pneumonia and COVID-19. Lower respiratory tract infections and COPD were, in 2019, in the list of the ten conditions more associated with long-term disabilities [24]. For COPD, in 2016, 251 million patients were diagnosed around the world with COPD, and predictions suggest that it will become the third most important cause of mortality by 2030 [25]. The amount and quality of the evidence, linking periodontitis and respiratory diseases, are however lower than the evidence already described for the associations with cardiovascular diseases and diabetes. However, a recent systematic review [9] and the consensus report of the joint Focused Workshop of the EFP with WONCA Europe [15] have concluded that, based on epidemiological evidence, a statistically significant association between periodontitis and COPD, obstructive sleep apnoea (OSA), and COVID-19 complications has been found. The evidence derived from intervention studies, however, was limited.

### Biological plausibility of the association periodontitis - systemic diseases

Periodontitis is an inflammatory disease, of infectious nature, since it is initiated by the accumulation of dental biofilms above and below the gingival margin, in which the initial inflammatory response may lead to microbial dysbiosis and, in some cases, to a chronic destructive immune-inflammatory response [26, 27].

The biological plausibility of the associations of periodontitis with different systemic conditions is based on, at least, four factors: (1) bacteraemia (live bacteria accessing the vascular system) of oral bacteria and periodontal pathogens; (2) increased systemic inflammation; (3) common genetic factors; and (4) common environmental risk factors.

In the association between *periodontitis and CVDs*, evidence is available to confirm the plausibility of the association based on the four elements listed:

1. People with periodontitis experience more frequent episodes of bacteraemia, as compared with periodontal health or gingivitis subjects, and these episodes may occur both during daily life activities, such as tooth brushing, flossing, or chewing, and after dental interventions, including professional prophylaxis, subgingival instrumentation, tooth extraction, third molar surgeries, or periodontal pocket probing [28]. Moreover, DNA from periodontal pathogens and even viable bacteria has been identified in atherothrombotic tissues [19, 28, 29]. Also, experimental pre-clinical models have demonstrated that these bacteria and/or their products and virulence factors influence the pathophysiology of atherosclerosis [30]. Finally, clinical studies have observed a dose-response relationship between the quantity and quality of the subgingival bacteria and vascular inflammation/subclinical atherosclerosis [31].
2. People with periodontitis present increased levels of systemic inflammation, as detected by inflammatory mediators that are associated with atherosclerosis, including high-sensitivity C-reactive protein (CRP) or interleukin (IL)-6 [30, 32].
3. Periodontitis and CVDs share numerous common genetic risk factors [33], hypothetically those favouring an exacerbated inflammatory response.
4. Periodontitis and CVDs share common environmental risk factors, being the most relevant example smoking habit [34].

For the association between *periodontitis and diabetes*, the proposed biological plausibility model also includes bacteraemia from the subgingival biofilm [28]. However, the studied mechanisms have mainly focussed on the effect of the elevated systemic inflammation with a dysregulated immune-inflammatory response [35, 36] and, specifically, on the hyperglycaemic state that may affect the periodontal tissues, both directly (e.g. with the formation of advanced-glycation end products, which interferes with periodontal wound healing [37, 38]), or indirectly, through the impact of the increased blood glucose on systemic inflammation and on the immune response (e.g. hyperglycaemia can negatively impact neutrophil function and T-helper cell response).

Regarding the association between *periodontitis and respiratory diseases*, two main mechanisms have been suggested: a direct mechanism, through the micro-aspiration of oral pathogens to the lower airways; and an indirect mechanism, through the systemic effect of bacteraemia and of the dumping of pro-inflammatory mediators produced in the periodontal tissues, that may affect the respiratory system, by favouring the onset and/or progression of other inflammatory conditions, including respiratory diseases [39].

### Association of periodontitis and COVID-19

As detailed in the “Methods” section, a recent systematic review explored both the epidemiological evidence and that derived from intervention studies, in the association of periodontitis and different respiratory diseases, including COVID-19 [9].

#### Epidemiological studies

A total of five publications, with a low risk of bias, were identified for the PECOS question, assessing epidemiological evidence: two cross-sectional studies [40, 41], two case-control studies [42, 43], and one cohort study [44] (see Table 1). The exposure variable (periodontitis) was defined by means of different clinical periodontal measures, including probing depth and clinical attachment loss, or by a combination of different clinical measures, using a variety of case definitions. In parallel, the diagnosis of SARS-CoV-2 infection was performed by virus detection in all five studies and, in addition, according to WHO guidelines in one study [43].

**Table 1:** Description of the selected studies [9], assessing the association of periodontitis and COVID-19 onset and severity. Some sentences are quoted directly from the original manuscripts

**Reference**	**Study design**	**Sample**	**Periodontitis diagnosis**	**Outcomes**	**Limitations**	**Conclusions**
Larvin et al. (2020) [40]	Cross-sectional study	*n* = 13,253 1,616 COVID+ (12%) 11,637 COVID- (88%).	Self-reported oral health indicators of painful or bleeding gums and loose teeth	Risk of COVID-19 infection in participants with painful or bleeding gums (OR=1.10, 95% CI [0.72; 1.69]) and loose teeth (OR=1.15, 95% CI [0.84; 1.59]), not significantly increased when compared to controls COVID-19 positive participants with painful or bleeding gums had a higher risk of mortality (OR=1.71, 95% CI [1.05; 2.72]) but not hospital admission (OR=0.90, 95% CI [0.59; 1.37])	A causal relationship cannot be established due to the cross-sectional design of the study. The use of self-reported oral health indicators as a surrogate for signs of PD could introduce bias, as research suggests self-reported PD prevalence is underestimated in populations	There was insufficient evidence to link PD with an increased risk of COVID-19 infection. COVID-19 patients showed significantly higher mortality if they have PD
Gupta et al. (2022) [41]	Cross-sectional study	82 COVID+ confirmed by nasopharyngeal swab testing	GR, GML, PPD, BOP, and number of teeth present/missing/carious were recorded. CAL was calculated	Higher severity of periodontitis led to 7.45 odds of requiring assisted ventilation, 36.52 odds of hospital admission, 14.58 odds of being deceased, and 4.42 odds of COVID-19-related pneumonia	A causal relationship cannot be established due to the cross-sectional design of the study. Another limitation can be the small sample size	Periodontitis seems to be related to poorer COVID-19-related outcomes
Anand et al. (2022) [42]	Case-control study	Cases: 79 PCR+ Controls: 71 PCR-	Plaque scores, calculus scores, tooth mobility, BOP, PPD, GR, CAL	Logistic regression analysis showed significant associations of mean plaque scores ≥ 1 (OR=7.01, 95% CI [1.83; 26.94]), gingivitis (OR=17.65, 95% CI [5.95; 52.37]), mean CAL ≥ 2 mm (OR=8.46, 95% CI [3.47; 20.63]), and severe periodontitis (OR=11.75, 95% CI [3.89; 35.49]) with COVID-19	As patients were not examined during the course of the disease, only patients who were willing to revisit the institution for the purpose of the study were available for data collection. During this process, valuable data from patients with severe forms of the infection may have been lost	It can be concluded that there is an association between periodontitis severity and COVID-19
Marouf et al. (2021) [43]	Case-control study	*n*= 568 COVID+ Cases: 40 experienced COVID complications Controls: 528 discharged without complications	The periodontal status was studied from posterior bitewings and panoramic radiographs in the patient’s electronic records	The risk of having COVID-19 complications in patients with periodontitis, after adjusting for possible confounders, was: OR=3.67 (95% CI [1.46; 9.27]) for all complications OR=8.81 (95% CI [1.00; 77.7]) for death OR=3.54 (95% CI [1.39; 9.05]) for ICU admission OR=4.57 (95% CI [1.19; 17.4]) for need of assisted ventilation	It does not address causality Using only one of the parameters (interdental bone loss) may limit the diagnostic accuracy	Periodontitis was significantly associated with a higher risk of complications from COVID-19, including ICU admission, need for assisted ventilation, and death and increased blood levels of markers linked worse COVID-19 outcome such as D-dimer, WBC, and CRP
Larvin et al. (2021) [44]	Cohort study	*n* = 58,897 14,466 (24.6%) were confirmed COVID+	Self-reported oral health indicators were used. Bleeding gums and painful gums were used as surrogates for mild to moderate PD, while self-reported loose teeth were indicative of severe PD	After adjustment for covariates, the risk for infection was not different in individuals with and without PD. The risk of hospital admission for people with PD was: 38% higher in overweight (HR=1.38, 95% CI [1.02; 1.87]) 124% higher in obese (HR=2.24, 95% CI [1.66; 3.03]) compared to those of normal weight The mortality rate in PD was 147% higher in participants who were obese (HR=2.47; 95% CI [1.61; 3.79])	The findings of this study were limited by the use of self-reported oral health indicators. As prevalence of PD is lower in the study sample, it is possible that the findings are subject to some selection bias and should be interpreted cautiously	Obesity had a more significant impact on infection and adverse COVID-19 outcomes than PD. The study revealed that PD may exacerbate the effect of obesity on hospitalization and mortality following COVID-19 infection

*PCR*, polymerase chain reaction; *OR*, odds ratio; *CI*, confidence interval; *HR*, hazard ratio; *ICU*, intensive care unit; *WBC*, white blood cells; *CRP*, C-reactive protein. *CAL*, clinical attachment level/loss; *GR*, gingival recession; *GML*, gingival marginal level; *PPD*, probing pocket depth; *BOP*, bleeding on probing; *PD*, "periodontal disease"

All studies, except one [40], reported associations between periodontitis and SARS-CoV-2 infection and/or its associated complications. In the retrospective cohort study [44], the risk for COVID-19 infection in individuals with periodontal self-reported symptoms was higher in participants who were overweight (odds ratio (OR)=1.21, 95% confidence interval (CI) [1.11; 1.32]) or obese (OR=1.37, 95% CI [1.23; 1.52]), than in participants of normal weight.

Different meta-analyses were performed: no association was found with COVID-19 infection (*n*=2, OR=3.45, 95% CI [0.36; 33.56], *p*=0.286), or with hospital admission (*n*=2, OR=5.76, 95% CI [0.15; 216.99], *p*=0.344). Conversely, associations were found with the need of assisted ventilation (*n*=2, OR=6.24, 95% CI [2.78; 13.99], *p*<0.001) and with COVID-19 associated mortality (*n*=3, OR=2.26, 95% CI [1.36; 3.77], *p*=0.002).

Due to its relevance, one of the studies selected [43] for the systematic review is described here in more detail. The study was possible due to the existence, in the State of Qatar, of the national electronic health records of Hamad Medical Corporation (HMC). This corporation provides public health and dental coverage to the entire country and includes 14 hospitals, and has a single electronic health record system (Cerner, Kansas City, USA), in which each patient retains a unique hospital identification number for both the medical and dental records. The availability of both medical and dental records allows for designing a case-control study, in which cases were COVID-19 patients who suffered complications (death, ICU admission, and/or mechanical ventilation), and controls those COVID-19 patients discharged without major complications. Only subjects older than 18 years, and with posterior bitewings and/or panoramic radiographs were included: interdental bone loss was measured in the posterior sextants using as reference the cement-enamel junction (CEJ) and the total length of the root. The percentage of bone loss was obtained from the most affected tooth using the criteria for staging of the 2018 classification of periodontitis [27]. The measurement of the exposure (by calibrated examiners) was, thus, defined as follows: periodontally healthy or initial periodontitis (stages 0–I), with bone loss less than the coronal third of the root length (<15%) in panoramic radiographs, or ≤2 mm in bitewing radiographs; and periodontitis (stages II–IV), with bone loss more than the coronal third of the root length (>15%) in panoramic radiographs, or >2 mm in bitewing radiographs. Analyses of 568 patients were adjusted for age, sex, smoking, body mass index (BMI), diabetes, and multiple co-morbidities. These adjusted models demonstrated a statistically significant higher risk for stages II–IV periodontitis, when compared with stages 0–I, for any complication (OR= 3.67, 95% CI [1.46; 9.27]), for death (OR= 8.81, 95% CI [1.00; 77.7]), for ICU admission (OR= 3.54, 95% CI [1.39; 9.05]), and need for assisted ventilation (OR= 4.57, 95% CI [1.19; 17.4]). In addition, laboratory biomarkers suggested a possible role for elevated levels of D-dimer, CRP, and white blood cell counts.

#### Intervention studies

No publications were found for the PICOS question formulated to identify evidence derived from intervention studies [9]. However, a very recent publication has addressed this issue [45], with the limitations of dealing with an acute condition, in which intervention studies most often have a retrospective nature.

The study was also designed as a case-control study [45], taking again advantage of the previously mentioned Qatar database, and with the same definition of cases and control, also previously explained [43]. The final sample was 1325 patients, with 71 considered as cases. The impact of periodontal status was explored, comparing periodontally healthy patients, and treated and untreated periodontitis patients. For the considered complications (death, ICU admission, mechanical ventilation, or any of them), the risk was higher for untreated periodontitis, followed by treated periodontitis and periodontally healthy. No statistically significant differences were observed between treated periodontitis and periodontally healthy, while a statistically significant higher risk for untreated periodontitis patients, when compared to those periodontally healthy, was observed for the complication: need for mechanical ventilation (adjusted OR=3.91, 95% CI [1.21; 12.57], *p*=0.022). The study also explored biological plausibility models and found that treated periodontitis patients had significantly lower levels of D-dimer and ferritin, in blood samples, than untreated periodontitis patients; and, also, that the detection of bacterial pulmonary infection (9 cases) was more frequent in periodontitis patients (5 out of 9), in which, complications were extremely frequent (7 out of 9, and 4 die) [45].

### Biological plausibility of the association periodontitis - COVID-19

Several hypotheses have been proposed to explain the observed associations, including translocation of periodontal pathogens and SARS-CoV-2 from the periodontal pocket to the bloodstream, and exacerbation of the cytokine storm via the low-grade chronic systemic inflammation [46].

The biological plausibility of the association of periodontitis with SARS-CoV-2 and COVID-19 can be explored from different perspectives:

- Following a similar approach as that considered for the association with other systemic diseases, with four components (bacteraemia, systemic inflammation, and common genetic or environmental risk factors). The advantage of this approach relies on its consistency with the evaluation of the association with other systemic diseases, and also it represents a comprehensive approach; conversely, the main disadvantage is that the approach may not be suitable for acute conditions, as COVID-19, and that lack of evidence is foreseeable for most items.
- Following a similar approach as that considered for the association with other respiratory diseases, with two components (micro-aspiration and systemic inflammation). The advantage of this approach relies on its consistency with the evaluation of the association with other respiratory diseases; conversely, the main disadvantage is that it is less comprehensive and, again, the approach may not be suitable for acute conditions, as COVID-19.
- A third approach would be to design a distinct approach, specific for COVID-19, combining the two previous approaches, and adding other possible factors relevant for COVID-19. The lack of consistency of the approach may be compensated by being better customized to assess a newly proposed association for a new condition.

Following the third approach, the so-called customized approach, the biological plausibility of the potential association of periodontitis with COVID-19 can be supported by the following facts:

1. SARS-CoV-2 can be detected in the gingival crevicular fluid (GCF) [47] and in periodontal pockets [48].
2. Periodontitis may favour increased transmission of SARS-CoV-2. This hypothesis may be explained by:

- The enrichment of angiotensin-converting enzyme 2 (ACE2) in the salivary glands and in the oral mucosa lining, including the sulcular epithelium and periodontal fibroblasts [49, 50].
- The ulceration in periodontal tissues (i.e. epithelium of the soft tissue wall), associated with periodontitis, may impair the protective role of epithelial cells, hence increasing the risk of SARS-CoV-2 [51].
- Subgingival epithelial cells in patients with periodontitis present increased levels of CD147, which is one of the receptors involved in SARS-CoV-2 transmission [52, 53].
- Periodontal inflammation has a boosting effect on the receptor expression [54].
- Periodontal pathogens might enhance SARS-CoV-2 virulence by cleaving its S glycoproteins [55].
- A prolonged exposure to *P. gingivalis* and other periodontal pathogens has been shown to induce an accelerated senescence in the lung alveolar epithelial cells [56], which may favour viral colonization and replication. Moreover, senescence would also promote bacterial adhesion to lung alveolar epithelial cells, leading to increase susceptibility to bacterial pneumonia [57].

3. Common risk factors and comorbidities. In a narrative review [58], Tamimi and co-workers elegantly analysed and listed common risk factors and comorbidities for both periodontitis (hypertension, obesity, age, diabetes, cerebrovascular disease, diabetes, cardiovascular diseases, chronic obstructive pulmonary disease, hypertension, atherosclerotic disease) and severe COVID-19 (hypertension, obesity, age, sex, diabetes, cardiovascular diseases, smoking, chronic pulmonary disease, coronary artery disease, chronic renal disease, cancer, atherosclerotic diseases), highlighting that most of them can behave as common comorbidities/risk factors.
4. Common genetic risk factors. Using the methodology of Mendelian randomisation, a research method using genetic variants to evaluate possible causal relationships between risk factors and disease outcomes, periodontal diseases have been significantly associated with susceptibility to suffer COVID-19 (OR=1.024, 95% CI [1.004; 1.045]; *p*=0.017) and with higher risk of hospitalization (OR=1.025, 95% CI [1.001; 1.049]; *p*=0.039).
5. Systemic inflammation in periodontitis. As with other common risk factors, in the same narrative review [58], different inflammatory biomarkers have been identified, which are elevated in both periodontitis (interleukins (IL) 1, 1β, 1RA, 2, 6, 7, 8, 9, 10, C-reactive protein (CRP), galectin-3, prostaglandin (PG) E2, interferon-gamma inducible protein 10, monocyte chemotactic protein-1, macrophage inflammatory protein-1α, fibroblast growth factor-2, granulocyte colony-stimulating factor, granulocyte-macrophage colony-stimulating factor, interferon-gamma, tumour necrosis factor (TNF)α, C3, C5, NOD-like receptor family pyrin domain-containing 3 inflammasome, ferritin) and in severe COVID-19 (IL 1, 1β, 1RA, 2, 6, 7, 8, 9, 10, CRP, galectin-3, PG E2, interferon-gamma inducible protein 10, monocyte chemotactic protein-1, macrophage inflammatory protein-1α, fibroblast growth factor-2, granulocyte-macrophage colony-stimulating factor, granulocyte colony-stimulating factor, interferon-gamma, TNF-α, C3 and C5, and NOD-like receptor family pyrin domain-containing 3 inflammasome, ferritin), highlighting their similarity, thus suggesting a common source from systemic inflammation. It has also been proposed [59] that inflamed periodontal tissues can act as reservoirs for pro-inflammatory cytokines, including TNF-α, IL-1α, IL-1β, and IL-6, exacerbating a previously existing systemic inflammation [60, 61]. In a recent narrative review, the authors concluded that periodontitis and COVID-19 have in common a “hyper-inflammatory” state [62].
6. Dysregulated immune response in periodontitis. With direct or indirect association with systemic inflammation, different biomarkers are found to be altered, both in severe COVID-19 and periodontitis [58], including coagulation biomarkers (elevated D-dimer, decreased fibrinogen, prolonged prothrombin time, decreased platelet counts, or increased plasminogen activator inhibitor), markers of immune cell activity (increased of neutrophils, of monocytes/macrophages, of release of neutrophil extracellular traps, of cluster of differentiation of T cells 4+, and of T helper 17 cells) or biomarkers of tissue-damage (matrix metalloproteinases, lactate dehydrogenase, alanine aminotransferase, troponin I, procalcitonin, aspartate aminotransferase). It has also been proposed [63] that patients with periodontitis show increased levels of different proteases (furin, cathepsin B, cathepsin L, cathepsin G), which may favour a more severe COVID-19, since they have been shown to increase the risk of complications in COVID-19 patients.
7. Association with respiratory diseases: micro-aspiration. Periodontitis may enhance pulmonary SARS-CoV-2 associated disease, since periodontal pathogens can be aspirated into the lower respiratory tract, inducing an overexpression of SARS-CoV-2 receptors [55]. Specifically, *Fusobacterium nucleatum* has been shown to upregulate ACE2 in the human respiratory epithelial cells in vitro [64]:

- It upregulates SARS-CoV-2 receptor angiotensin-converting enzyme 2 in alveolar epithelial cells.
- It induces interleukin (IL)-6 and IL-8 production by alveolar epithelial cells.
- It induces IL-6 and IL-8 expression by bronchial and pharyngeal epithelial cells.

In this context, patients with mild COVID-19 frequently aspirate periodontopathic bacteria, so SARS-CoV-2 infection is promoted, and inflammation in the lower respiratory tract may become more severe in the presence of viral pneumonia.

## Clinical relevance

Due to the possible association between periodontitis, and an increased susceptibility and severity for COVID-19, additional efforts should be made to improve oral and periodontal health, with the promotion of healthy lifestyles, including adequate oral hygiene habits.

## Conclusions

The evidence discussed in the present narrative review may suggest the following conclusions:

- The association between periodontitis and different systemic conditions has been established, as well as its biological plausibility.
- Initial evidence suggests that periodontitis may be associated with a more severe COVID-19 and with a higher risk of death due to COVID-19.
- Oral hygiene and periodontal health should be considered as part of a healthy lifestyle and they are very relevant for public health policies.
